# Pancreatic PYY but not PPY expression is responsive to short-term nutritional state and the pancreas constitutes the major site of PYY mRNA expression in chickens

**DOI:** 10.1016/j.ygcen.2017.07.002

**Published:** 2017-10-01

**Authors:** Angus M.A. Reid, Peter W. Wilson, Sarah D. Caughey, Laura M. Dixon, Rick B. D'Eath, Victoria Sandilands, Timothy Boswell, Ian C. Dunn

**Affiliations:** a Roslin Institute, University of Edinburgh, EH25 9RG, Scotland, United Kingdom; b Scotland’s Rural College (SRUC), Edinburgh EH9 3JG, Scotland, United Kingdom; c SRUC Avian Science Research Centre, Auchincruive, KA6 5HW, Scotland, United Kingdom; d School of Biology, Newcastle University, NE1 7RU, England, United Kingdom

**Keywords:** Peptide YY, Pancreatic polypeptide, Energy homeostasis, Feeding, Broiler

## Abstract

PP-fold peptides such as peptide YY (PYY) and pancreatic polypeptide (PPY) are known to play key roles in vertebrate energy homeostasis. Until recently, no gene sequence was available for avian PYY and therefore a gap in knowledge of regulation of its expression exists in avian species. Here we further evidence the mRNA sequence for chicken PYY and show that the pancreas is the major site of its mRNA expression, with a secondary peak of expression around the distal jejunum, in contrast to mammals where the large intestine is the major site of PYY expression. We also demonstrate that pancreatic PYY expression is responsive to short-term and long-term nutritional state, increasing within hours of feeding, in contrast to intestinal PYY which does not fluctuate to the same extent, and pancreatic PPY which appears to be primarily determined by long-term energy state. Both pancreatic PYY and PPY expression were found to exhibit ontogeny, being evenly distributed throughout the pancreas in young (2wk) chicks but having a decreasing splenic to duodenal gradient by adolescence (12wk).

## Highlights

• Chicken and quail PYY mRNA sequences evidenced.
• Pancreas identified as the major site of PYY mRNA expression in chickens.
• Peak of intestinal expression evidenced around the distal jejunum.
• Ontogenic development of gradient pancreatic expression of chicken PYY and PPY reported.
• Chicken pancreatic PYY shown to increase in response to short-term nutritional state.

## 1 Introduction

Peptide YY (PYY) is one of three known members of the PP-fold family of proteins, along with neuropeptide Y (NPY) and pancreatic polypeptide (PPY) (Cerda-Reverter and Larhammar, 2000). The structure and function of the PYY gene is relatively well-documented in mammals (Batterham and Bloom, 2003, McGowan and Bloom, 2004, Ueno et al., 2008); however, very little transcriptional work has been reported in avian species. This is due to the lack of a gene sequence, despite early elucidation of the peptide sequence (Conlon and Oharte, 1992) and relatively high conservation of the peptide (Blomqvist et al., 1992). The most recent chicken genome build – Gallus_gallus-5.0, GenBank accession GCF_000002315.4 – erroneously annotated a predicted PYY gene sequence at position KQ759583.1: 13,290–14,841 (Ensembl) but this is in fact the chicken PPY gene, encoding pancreatic polypeptide (Nata et al., 1993). The first articles evidencing the true chicken PYY mRNA sequence were recently published (Aoki et al., 2017, Gao et al., 2017) but these disagree on the transcriptional start and termination sites. Issues in definitively characterising the transcript may be due to homology with the other PP-fold gene family members and regions of high GC content, a feature of PYY genes across taxa (e.g. human PYY mRNA (NM_004160.5) region 940–1020 = 77.8% GC content, lizard PYY (XM_003222643.3) region 400–444 = 71.1% GC).

In vertebrates, peripheral PYY is purported to act as a satiety factor released from the gastrointestinal tract after feeding to curb appetite via afferent vagal Y-receptors or directly within the arcuate nucleus of the hypothalamus (Batterham and Bloom, 2003, Batterham et al., 2002, Simpson et al., 2012, Ueno et al., 2008). PYY-expressing neurones also exist in the brain in mammals and fish, however some contention exists as to the relative levels of PYY expression in different tissues and their functional significance. In goldfish for example, central PYY is reported to be more highly expressed than intestinal PYY and is upregulated in satiety, in contrast to observations for other vertebrates including other fish (Murashita et al., 2009, Wall and Volkoff, 2013). Discrete populations of PYY-expressing cells have been identified throughout the brain (Bottcher et al., 1985, Cerda-Reverter et al., 2000) and digestive system (Ekblad and Sundler, 2002, El-Salhy et al., 1983), so opposing or unrelated roles for PYY expressed from distinct regions might account for inconsistencies in whole-tissue measurements, along with interspecific variation and differences in satiety state. In tetrapods at least, the accepted dogma is that peripheral PYY acts as an anorectic factor released from the intestine after meals to curb food intake and modulate gastrointestinal function, whereas central PYY has a functionally opposite orexigenic effect (McGowan and Bloom, 2004, Ueno et al., 2008).

Distinct binding preferences and tissue distributions of several Y-receptors (Dumont et al., 1998, Keire et al., 2002, Larhammar, 1996) allude to the broad range of biological functions attributed to central and peripheral PP-fold peptide activity. Discrete ligand expression and variable receptor specificity conferred by proteolytic processing of ligands (Cerda-Reverter et al., 2000, Mentlein et al., 1993) likely further facilitate diverse and dynamic endocrine and paracrine roles for PP-fold peptides *in vivo*. Chicken PYY is known to differ in primary structure to mammalian PYY, resulting in anomalous cleavage of the signal peptide and an elongated 37-residue ligand apparently impervious to proteolysis by DPP4 (Conlon and Oharte, 1992). This theoretically restricts posttranslational variability of receptor specificity and implies the possibility of alternative modes of PYY action in the chicken (and perhaps across avian species) but the biological implications of proteolysis are not comprehensively understood for any species. Since chicken PYY remains under-studied, describing the tissue distribution and nutritional state-responsiveness of its expression is a priority to inform future studies investigating the precise biological roles of PYY in birds.

PPY was originally identified in the chicken (Kimmel et al., 1975) and is known to be predominantly expressed in the pancreas in vertebrates (Ekblad and Sundler, 2002, Gao et al., 2017). PPY is relatively well-characterised as a satiety hormone, with increased circulating PPY observed within minutes to hours after feeding in avian (Johnson and Hazelwood, 1982) and mammalian (Asakawa et al., 2003) models. Exogenous PPY was also shown to reduce food intake in a dose-dependent manner after intraperitoneal administration in mice (Asakawa et al., 2003), however no such peripheral injection studies have been carried out for avian PPY.

Defining the roles of PP-fold family members is important for understanding energy balance and growth in vertebrate species. This includes domestic fowl, for which there is a need to understand appetite regulation so that management of birds used for human food production might be optimised to maximise efficiency and ameliorate welfare concerns, such as restricted feeding in breeding meat-type birds (De Jong and Guemene, 2011). The few studies carried out on avian PYY have mostly involved application of exogenous PYY peptide and demonstrated an orexigenic effect of centrally-administered PYY (Ando et al., 2001, Kuenzel et al., 1987) and digestion- and growth-regulating effects of PYY applied systemically *in ovo* (Coles et al., 2001, Coles et al., 1999). Recent results from peripheral PYY administration and transcriptional measurements in feeding studies support the conserved role of intestinal PYY as an anorectic satiety factor in chickens (Aoki et al., 2017). Although PYY is traditionally considered an intestinal peptide, there is growing evidence that pancreas-derived PYY is important in regulation of digestion and satiety. Interesting ontogenic and regional gradient patterns of pancreatic expression of PYY and PPY have been demonstrated in mammals (Ekblad and Sundler, 2002, El-Salhy et al., 1983, Sandström and El-Salhy, 2002). Functional regulation of PYY expression has not yet been described in birds. In order to improve knowledge of peripheral control of satiety in birds and enable comparisons to be drawn when establishing conserved and divergent roles for PP-fold family peptides across taxa, we investigate in this study the tissue distribution of PYY and PPY mRNAs in the chicken and the effects of nutritional state on their expression, particularly in the pancreas.

## 2 Materials and methods

### 2.1 Sequence derivation

#### 2.1.1 Avian PYY and PPY

The large amount of high-throughput sequencing information from the chicken (*Gallus gallus*) available in the sequence read archive (SRA) (Leinonen et al., 2011b) was mined to derive a contiguous mRNA sequence for chicken PYY. Relevant experiments were identified by using appropriate search terms at the European Nucleotide Archive (Leinonen et al., 2011a). Data files were then probed for PYY mRNA sequence using the known chicken PYY peptide sequence (AAB24283.1) as a query in tblastn (NCBI). Short read sequences of interest were downloaded in FASTA format and read into GAP (Bonfield and Whitwham, 2010) for alignment. The process was iterative and as each new consensus was built the SRA files were re-interrogated using nucleotide BLAST until no further 3′ or 5′ extension was obtained. The final consensus sequence was analysed for open reading frames using ExPasy Translate (Gasteiger et al., 2003) and likely signal peptide cleavage sites using SignalP (Petersen et al., 2011).

For confirmation of the cDNA 5′ end in chicken, rapid amplification of cDNA ends (RACE) was completed using the Roche 2nd generation 5′/3′ RACE kit as per the manufacturer’s protocol and LightRUN Sanger sequencing (GATC Biotech) was employed to sequence the product. A similar data-mining process was followed for quail (*Coturnix coturnix*) PYY. This was only to increase confidence in inference to the evolving structures of vertebrate PYY and no further characterisation was carried out for quail PYY. The chicken PPY gene sequence was already definitively known (NM_204786.1) (Nata et al., 1993).

### 2.2 Animal experiments

Each animal experiment was approved by the Roslin Institute Animal Welfare and Ethical Review Body or SRUC Animal Ethical Committee, and compliant with UK Home Office legislation.

#### 2.2.1 Distribution of expression of PYY and PPY across chicken tissues

In order to assess the distribution of expression of PYY and PPY in chicken tissues, four Ross 308 broilers raised in standard conditions were killed at 42 d and a broad range of tissue samples was collected.

#### 2.2.2 Effects of short-term nutritional state on expression of PYY and PPY

Chicks from the 22nd generation of a pedigree broiler-layer hybrid line were reared under standard lighting (14 L:10 D) and temperature (26 °C ambient) conditions in one pen with *ad libitum* access to food until 10 days of age when they were separated into two experimental group pens (n = 12 per group) to balance sex and family. *Ad libitum* access to food was maintained until removal of food from both groups at 14 days of age, 2 h before lights-on. This was followed either by reintroduction of food after 3.5 h (*ad libitum* group, AL) or by maintenance of fasting conditions for a further 7–8 h to give a total fast duration of 10.5–11.5 h (fasted group, F). At the experimental endpoint, birds were killed by cervical dislocation and 40–100 mg samples from the pancreas head (splenic end) and tail (duodenal end) were immediately collected and snap-frozen on dry ice.

#### 2.2.3 Effects of long-term nutritional state on expression of PYY and PPY at several timepoints after feeding

The general approach in testing different dietary regimes was based on a previously-described study (Dunn et al., 2013b). Broiler breeders reared under standard conditions in floor pens were fed on a standard commercial restricted dietary regime until six weeks of age and were then assigned to one of three dietary treatments in a replicated design by ranked randomisation to balance bodyweight. One group was released from restriction and given *ad libitum* access to food, whilst the other two were maintained on restricted diets. One of the food-restricted groups (Ram) was fed daily at 07:00 (lights-on) and the other (Rpm) at 16:00 (one hour before lights-off) and all restricted pens routinely consumed their entire daily ration within 10–20 min of feeding. Birds were killed by barbiturate overdose at 12 weeks of age, at 1, 7, 16 or 22 h after feeding (culling began at these timepoints but took up to two hours to complete) in an order balanced by treatment group (n ≥ 14 per timepoint per treatment). Tissue samples were collected from a central area of the pancreas and the small intestine proximal to the vitelline diverticulum and snap-frozen in liquid nitrogen.

#### 2.2.4 Roles of gut fill and nutrient uptake

Broiler breeders were reared under standard conditions in floor pens with commercial food restriction to achieve the breeding company’s performance objectives (Aviagen, 2007) until 11 weeks of age when birds were ranked and randomised on the basis of body weight into one of three experimental groups, evenly distributed among individual cages in 3 replicate rooms. Following a 5-day cage acclimatisation period, birds were fed either *ad libitum* (AL, n = 24), continued commercial restriction (FR, n = 24) or commercial restriction plus 15% (w/w) dry ispaghula husk powder, (IH, n = 24) for a further 2.5 d before cull by anaesthetic overdose. Samples of 40–100 mg were taken from the pancreas and snap-frozen on dry ice. Individuals from each treatment were evenly distributed between and within each dissection day and processing batch to avoid confounding bias. Groups were balanced for body weight, and sex was taken into account in statistical analyses. Material from a later replicate experiment, excluding the ispaghula husk treatment, was harvested for *in situ* hybridisation.

### 2.3 Design of oligonucleotide primers and probes

**Fig. 1:**
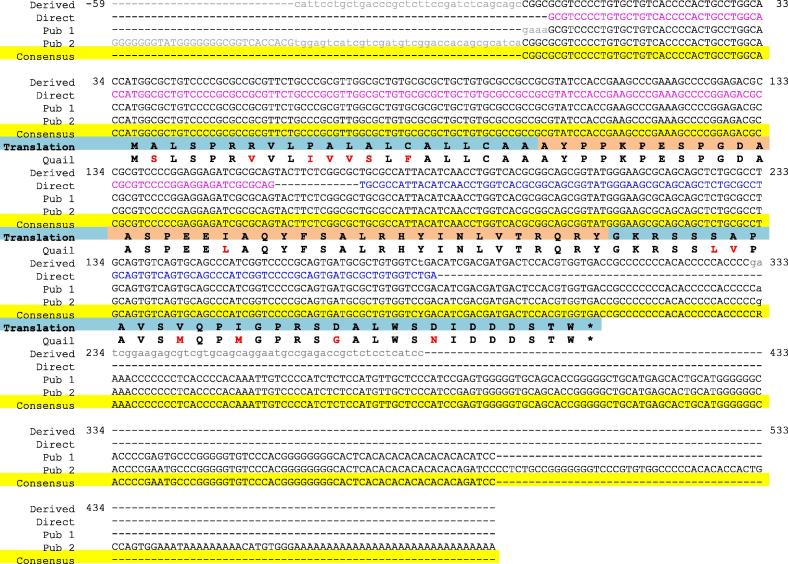
Collated chicken PYY cDNA sequence information. Derived chicken PYY sequence information (‘Derived’, see section 2.1.1.) is aligned with sequence evidenced directly in the present study (‘Direct’), data from Aoki et al. (‘Pub 1’) and data from Gao et al. (‘Pub 2’) and a consensus sequence reported. Bases evidenced by direct sequencing are coloured pink (chicken 5’RACE product, see section 2.1.1.) or blue (qPCR amplicon, see sections 2.3. & 2.5.). Lower case letters mark sites of disagreement between sources. Grey letters denote poorly evidenced bases. Black letters denote bases consistent with the reported consensus. Hyphens represent unknown residues. Standard IUPAC nucleotide codes have been employed where ambiguity in the consensus exists. The translated amino acid sequence is highlighted blue, with the mature peptide PYY_1-37_ highlighted orange and the derived quail PYY translated product is aligned, with discrepancies between species highlighted in red.

See Table 1 for details of primers and probes used in this study. Novel primers were designed using Primer 3 (Rozen and Skaletsky, 2000, Untergasser et al., 2012) to amplify chicken PPY (NM_204786.1), 14-3-3 protein zeta (YWHAZ) (NM_001031343.1), NADH:ubiquinone oxidoreductase subunit A1 (NDUFA1) (NM_001302115.1) and derived chicken PYY (Fig. 1) mature mRNA sequences. Primers for quantification of lamin B receptor (LBR) as a reference gene were previously described (Dunn et al., 2013a). All oligonucleotides were sourced from Sigma-Aldrich (UK).

**Table 1:** Details of oligonucleotide primers and probes.

Oligonucleotide name	Type	Sequence (5′-3′)	qPCR target accession & amplicon length	Application(s)
PYY-ARF1	Primer	TTACATCAACCTGGTCACGC	N/A 112bp	PCR
PYY-ARR3	Primer	TCAGACCACAGCGCATCACT		PCR, 5′RACE
PPY 02 Primer F	Primer	TCTACAACGACCTCCAGCAG	NM_204786.1 86bp	PCR
PPY 03 Primer R	Primer	CTCTTCGCACAGCACCCG		PCR
PYY-GSP2	Primer	GATGGGCTGCACTGACACT	–	5′RACE
PYY-GSP3	Primer	TGACCAGGTTGATGTAATGGC	–	5′RACE
YWHAZ_F	Primer	GTGGAGCAATCACAACAGGC	NM_001031343.1 223bp	PCR
YWHAZ_R	Primer	GCGTGCGTCTTTGTATGACTC		PCR
LBR-F	Primer	GGTGTGGGTTCCATTTGTCTACA	NM_205342.1 80bp	PCR
LBR-R	Primer	CTGCAACCGGCCAAGAAA		PCR
AR_PYY-ISH1	Probe	TGCTGCGCTTCCCATACCGCTGCCGCGTGACCAGGTTGATGTAAT	–	ISH
AR_aPP_ISH1	Probe	GTGACCACGTTGAGGTACTGCTGGAGGTCGTTGTAGAAGCGGATG	–	ISH

### 2.4 Preparation of cDNA

Total RNA was isolated from tissue homogenised in TRIzol reagent (Invitrogen) using the Direct-zol RNA Kit (Zymo Research) to manufacturer’s specifications, including in-column DNase treatment. Reverse transcription of 1 μg total RNA per sample was performed using the High Capacity Reverse Transcription Kit (Applied Biosystems) according to manufacturer’s guidelines and the product volume adjusted to 110 μl per sample.

### 2.5 Quantitative polymerase chain reaction (qPCR)

All qPCR assays employed Brilliant III Ultra-fast SYBR Green qPCR Mastermix and the Mx3005p qPCR System with MxPro software (Agilent Technologies) according to the manufacturers’ guidelines and as described previously (Whenham et al., 2015). Briefly, 10 μl SYBR mix, 8 μl cDNA product, 0.4 μl 20 μM forward primer, 0.4 μl 20 μM reverse primer, 0.3 μl 1/500 rox reference dye solution and 0.9 μl H_2_O were mixed for each 20 μl reaction. Thermal conditions were consistent for all assays: 50 °C; 120 s, 95 °C; 120 s, (40 cycles of 95 °C; 15 s, 60 °C; 30 s), then 95 °C; 60 s, 60 °C; 30 s, 95 °C; 15 s. Apparent reaction efficiency ranged between 90–105%, as determined by analysis of the standard dilution curve. Samples were distributed between and across plates to avoid confounding bias from plate effects. Each assay was developed by selection from several possible primer pairings; a mock qPCR reaction subjected to the aforementioned qPCR thermal profile was run for each prospective primer pair. Normal PCRs using Faststart reagents (Roche) and a standard PCR thermal profile (95 °C; 240 s, (40 cycles of 95 °C; 30 s, 58 °C; 30 s, 72 °C; 30 s), then 72 °C; 420 s) were also run for each prospective pair. Paired qPCR and normal PCR products were analysed together by visualisation after agarose gel electrophoresis. For each gene, the highest-amplifying reaction lacking visible primer-dimer and infidelity signals determined selection of the primer pair to be used for qPCR. Amplicons from the selected primer pairings’ normal (Faststart) PCRs were isolated using the QIAquick Gel Extraction Kit (Qiagen) and bidirectionally sequenced using LightRUN Sanger sequencing (GATC Biotech) to confirm identity. Standard curve samples of known concentration were prepared by nanodrop measurement and serial dilution of these stock amplicon samples. LBR, YWHAZ and NDUFA were chosen as reference genes due to their reliability in previous avian studies (Olias et al., 2014) and quantified as above. Normalisation was achieved by using the geometric mean of some combination of LBR, YWHAZ and NDUFA expression values, or the LBR value alone where YWHAZ and NDUFA were not measured, as a division factor for the gene of interest expression value. For the expressional distribution analysis (Section 3.2), differences in transcriptional activity between tissues (which could adversely affect the quantitation of gene expression) were accounted for by using the yield of RNA per mg tissue as a multiplication factor for genes of interest and reference genes prior to comparison.

### 2.6 *In situ* hybridisation

All *in situ* hybridisation was performed using a standardised protocol and reagents as described previously (Meddle et al., 2007). Briefly, purified radiolabelled (^35^S dATP-tailed) oligonucleotide probes specific to mRNAs of interest (see Table 1) were incubated overnight with fixed 15 μm tissue sections on polylysine-coated slides. Slides were then exposed in autoradiographic emulsion for 14 days before development and fixation and subsequently counterstained with haemotoxylin and eosin.

### 2.7 Statistical analyses

One-way or two-way ANOVA was initially employed to ascertain statistical significance of differences between group means. Log transformation was used to approximate normality of residual values where necessary. Where residual values could not be approximated to normal by log transformation, the non-parametric Kruskal-Wallis H test was used. Where appropriate, variables were used as blocking factors in analyses and balanced across batches to avoid introduction of confounding biases. Results for ANOVA are presented as the F-ratio (F), with subscript information on the degrees of freedom between and then within means, and the probability value (p). Differences between individual groups were assigned using least-significant differences. Results for the H test are presented as the H statistic (H) and probability value (p). Spearman’s rank correlation coefficient was determined for suspected correlative relationships, with results presented as the rho value (r_s_), with degrees of freedom in brackets, and the exact probability value (p). Probability values of 0.05 or less were considered statistically significant. All statistical analyses were performed using Genstat 13 (VSN International).

## 3 Results

### 3.1 Sequence information

#### 3.1.1 Chicken and quail PYY

Files with successful outcomes for chicken preproPYY and quail preproPYY are listed in Table 2 and were principally from intestine and brain samples. The mRNA sequence derived from assembly of short reads for chicken preproPYY included the entire putative translated region (90aa), the stop codon, 74bp of putative 5′ untranslated region (UTR) and 73bp of putative 3′ UTR (Fig. 1). Sequencing of the chicken PYY 5′ RACE product and qPCR amplicon (submitted to GenBank, accessions MF455302 & MF455303) confirmed the sequence of the translated region and suggested that the 5′ UTR is 38bp. The assembly of short reads for quail preproPYY mRNA included the entire putative translated region and short extensions into the 5′ and 3′ UTRs but only the translated product is reported (Fig. 1).

**Table 2:** SRA experiments with successful outcomes for chicken and quail PYY. Accession numbers listed accord with the SRA database. All studies employed an RNA-seq strategy and the source materials for all experiments were transcriptomic. Sequencing was of complementary DNA (cDNA) or RNA directly (polyA/random PCR). The layout column indicates whether unidirectional (single) or bidirectional (paired) sequencing evidenced each read. Where possible, acknowledgement of contributions to the SRA is made by citation of published articles resulting directly from these experiments.

SRA accession	Species	Instrument	Source & selection	Layout	Reference
ERP006915	Chicken	Illumina Genome Analyzer IIx	Transcriptomic, cDNA	Single	Smith et al. (2015)
SRP015997	Chicken	Illumina HiSeq 2000	Transcriptomic, cDNA	Paired	Barbosa-Morais et al. (2012)
SRP018692	Chicken	Illumina Genome Analyzer II	Transcriptomic, cDNA	Single	Connell et al. (2012)
ERP011662	Quail	Illumina HiSeq 2500	Transcriptomic, cDNA	Paired	–
ERP012532	Quail	Illumina HiSeq 2500	Transcriptomic, cDNA	Single	–
SRP067215	Quail	Illumina HiSeq 2500	Transcriptomic, polyA	Paired	–
SRP071654	Quail	Illumina HiSeq 2000	Transcriptomic, random PCR	Single	–

### 3.2 Distribution of expression of PYY and PPY across chicken tissues

**Fig. 2:**
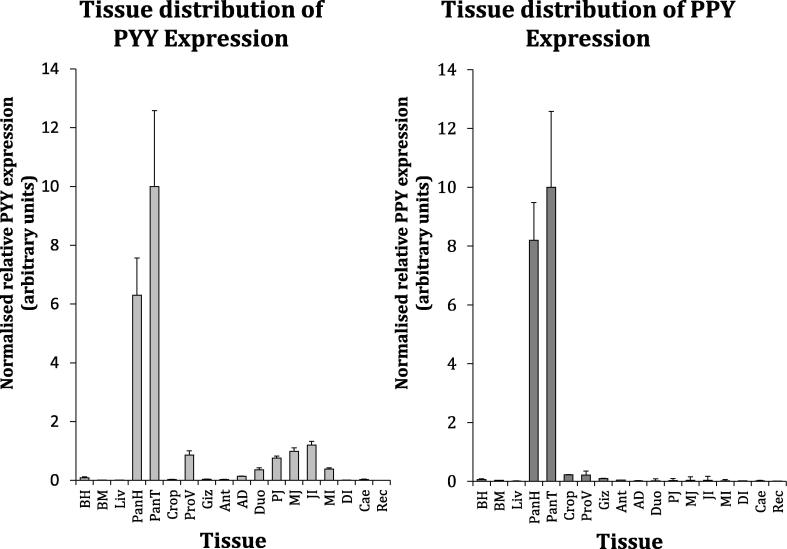
Tissue distribution of chicken PYY and PPY. Normalised relative mean (±SEM) PYY and PPY mRNA expression for 18 tissue types in six-week-old male Ross 308 broilers (n = 4): basal hypothalamus (BH), breast muscle (BM), liver (Liv), pancreas duodenal end (head; PanH), pancreas splenic end (tail; PanT) crop, proventriculus (ProV), gizzard (Giz), antrum (Ant), antro-duodenal boundary (AD), duodenum (Duo), proximal jejunum (PJ), mid-jejunum (MJ), jejuno-ileal boundary proximal to the vitelline diverticulum (JI), mid-ileum (MI), distal ileum (DI), caecum (Cae) and rectum (Rec).

**Fig. 3:**
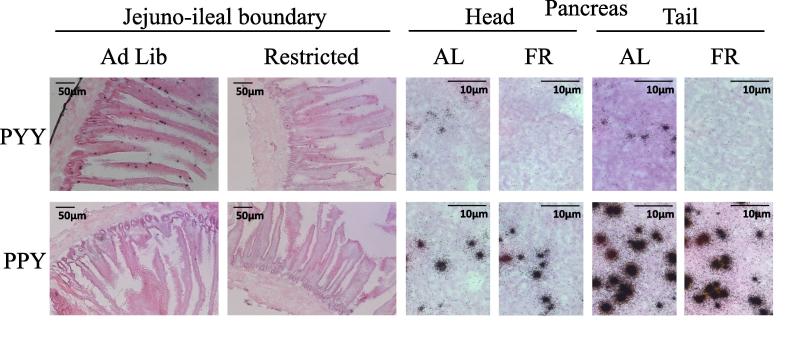
*In situ* hybridisation of 15 μm tissue sections. Jejuno-ileal boundary (JI), pancreas head (duodenal end) and pancreas tail (splenic end) tissue sections from two adolescent (12wk) broiler breeders, demonstrating expression of PYY (upper panels) and PPY (lower panels) by hybridisation with gene-specific oligonucleotide probes (see Table 1).

Profiles of the distributions of PYY and PPY in chicken across several tissues are detailed in Fig. 2. Of particular note, we observed that the pancreas is the major site of PYY transcription and that the major site of gastrointestinal PYY expression is around the distal jejunum. High pancreatic compared to intestinal expression was clear upon scrutiny of *in situ* hybridised sections (Fig. 3), which also confirmed that PPY is expressed at a considerably higher level than PYY in the chicken pancreas but is not obviously expressed in the intestine. Interestingly, the pancreas yielded more RNA than any other tissue per unit weight, and yield differed significantly between tissue types (F_17,51_ = 4.69, p < 0.001).

### 3.3 Functional expression

#### 3.3.1 Regional pancreatic distribution of PYY and PPY expression and the effect of short-term nutritional state

**Fig. 4:**
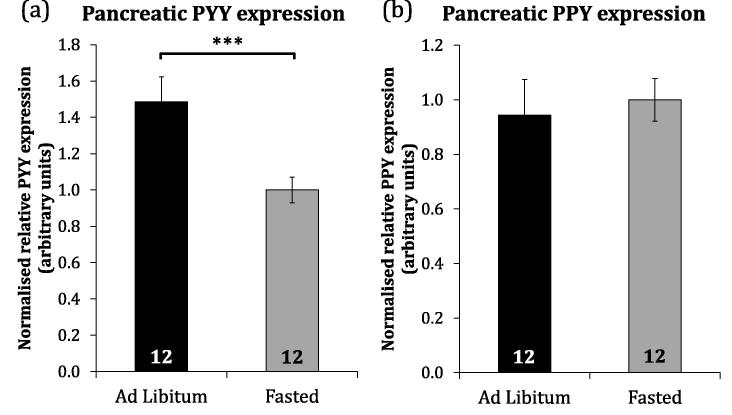
Pancreatic PYY but not PPY expression responds to short-term nutritional state. Normalised relative mean (±SEM) PYY (a) and PPY (b) expression in the pancreas of satiated (*ad libitum* fed) and fasted (11 h fast) broiler-layer hybrid birds at 2 weeks of age. The number of individuals in each group is indicated within bars. Statistical significance is indicated (^***^ANOVA p < 0.001).

Neither pancreatic PYY (F_1,22_ = 0.02, p = 0.898) nor PPY (F_1,22_ = 0.15, p = 0.706) expression was dependent on the region of the pancreas sampled in young chicks (2wk) but both were greater in the pancreas splenic end than the duodenal end in adolescent birds (12wk) (PYY, F_1,7_ = 13.03, p = 0.009; PPY, F_1,7_ = 6.57, p = 0.037). PYY expression was positively correlated with PPY expression in both 2wk (r_s_ (45) = 0.506, p < 0.001) and 12wk (r_s_ (14) = 0.782, p < 0.001) birds. PYY expression was responsive to short-term nutritional state independent of sex, being significantly higher in the pancreas of satiated (AL) birds compared to those experiencing short-term food restriction (F) (H = 0.768, p = 0.006) (Fig. 4a) however pancreatic PPY expression did not differ between these groups (H = 0.75, p = 0.386) (Fig. 4b). Results in this section were corroborated by *in situ* hybridisation (Fig. 3).

#### 3.3.2 Effect of long-term nutritional state on expression of PYY and PPY at several timepoints after feeding

The three dietary groups in the long-term nutritional experiment were balanced for bodyweight at six weeks of age (see Section 2.2.3) but the average bodyweight for the group fed *ad libitum* (AL, 3065 ± 65 g) was over double those of the morning-fed restricted (Ram, 1202 ± 18 g) and evening-fed restricted (Rpm, 1215 ± 14 g) groups at cull (12wk).

**Fig. 5:**
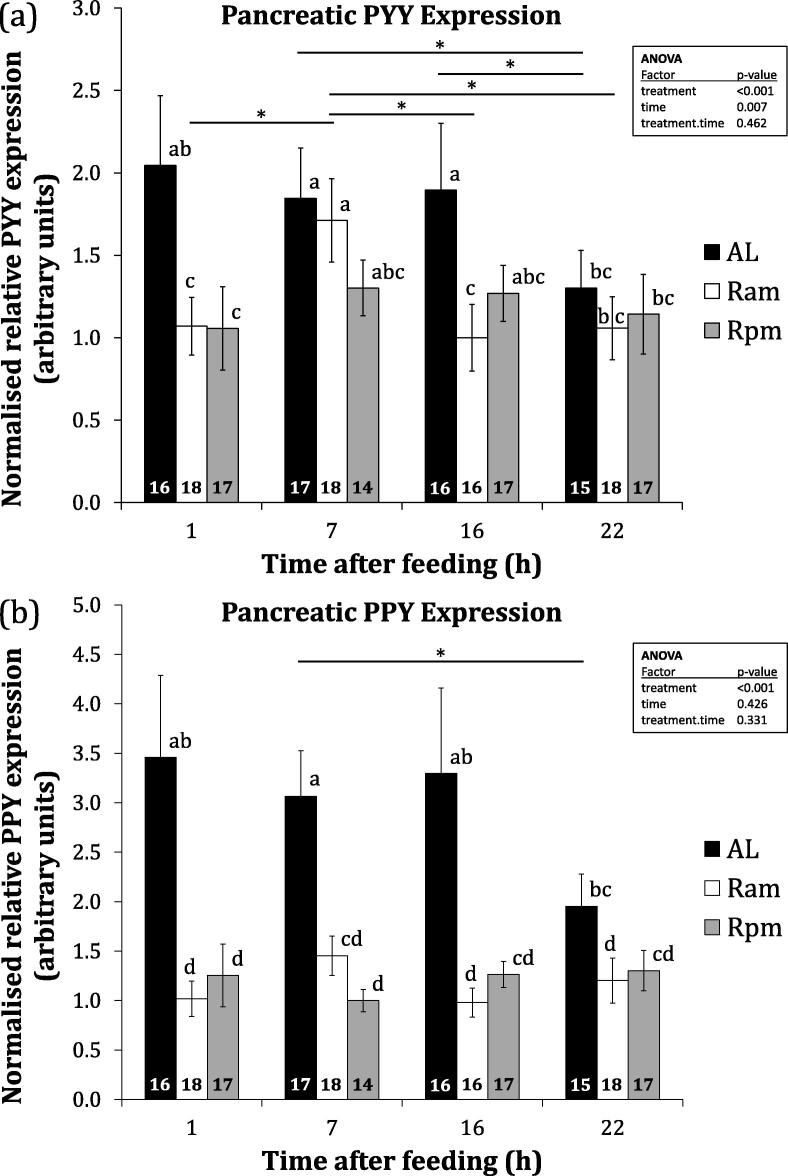
Effects of long-term nutritional state on expression of PYY and PPY at several timepoints after feeding. Normalised relative mean (±SEM) pancreatic PYY (a) and PPY (b) expression in broiler breeder chickens fed chronic *ad libitum* (AL), restricted morning ration (Ram) or restricted evening ration (Rpm) diets. The number of individuals in each group is indicated within bars. Statistical tests were performed on log-transformed data, but observed data are presented. Data points not labelled with a common letter are statistically significantly different at P < 0.05. Statistically significant differences across sampling points within groups are indicated (^*^p < 0.05).

The major experimental variables were dietary treatment (treatment) and time after feeding (time). Some gene expression analyses revealed a significant treatment by time interaction (treatment time). Results of a preliminary assay on a treatment- and time-balanced subset of samples from the long-term nutritional state experiment (see Section 2.2.3) showed that pancreatic expression of PYY was indeed statistically significantly higher than intestinal expression across all treatment groups and time-points (F_1,47_ = 175.1, p < 0.001). Intestinal PYY expression did not exhibit fluctuation throughout the day (F_3,33_ = 1.04, p = 0.387) comparable to that seen for pancreatic PYY expression, which did fluctuate through the day in the same subset (F_3,33_ = 5.28, p = 0.004). There was also a significant treatment by time interaction (F_6,33_ = 3.29, p = 0.012) for pancreatic PYY expression, whereas intestinal PYY expression exhibited no such interaction (F_6,33_ = 1.13, p = 0.368). Pancreatic and intestinal PYY expression were not obviously correlated (r_s_ (47) = 0.129, p = 0.097). These results informed a subsequent exclusion of intestinal samples and the pancreas was then analysed in a greater number of individuals (Fig. 5).

Pancreatic PYY differed significantly between treatments (F_2,176_ = 8.69, p < 0.001) and timepoints (F_3,176_ = 4.18, p = 0.007) when all data are analysed together. Several significant differences in pancreatic PYY expression between timepoints within groups were detected for AL and Ram, but not Rpm treatments (Fig. 5a). Pancreatic PPY differed significantly between treatments (F_2,176_ = 29.74, p < 0.001), but not timepoints (F_3,176_ = 0.93, p = 0.426) when all data are analysed together. The only difference detected between timepoints within a treatment was between AL individuals 7 h and 22 h after feeding (Fig. 5b).

#### 3.3.3 Roles of gut fill and nutrient uptake

**Fig. 6:**
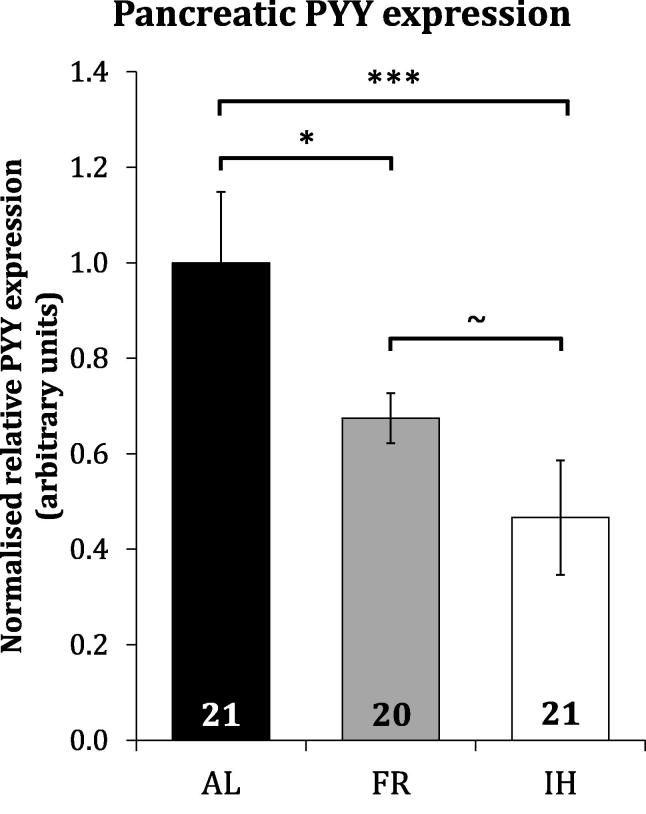
Roles of gut fill and nutrient uptake on Pancreatic PYY expression. Normalised relative mean pancreatic PYY expression in 12-week-old broiler breeder chickens subject to short-term *ad libitum* fed (AL), restricted (FR) or diluted (isocaloric restriction with 15% (w/w) ispaghula husk) (IH) diets for 2.5 days. The number of individuals in each group is indicated within bars. Statistical significance by one-way ANOVA is indicated (∼p < 0.07, ^*^p < 0.05, ^***^p < 0.001).

Pancreatic PYY expression was significantly reduced in broiler breeders under long-term food restriction versus those re-fed *ad libitum* for 2.5 days and lower in food-restricted birds with additional dietary fibre, compared to those without, to an extent approaching statistical significance (Fig. 6).

## 4 Discussion

The close agreement of our 5′ RACE product sequencing with 2017 data from Aoki et al. provides convincing evidence that the transcriptional start site (TSS) for chicken PYY is approximately 38bp upstream of the translational start codon. It was noted that the 5′ extremity of the sequence published by Gao et al. (Gao et al., 2017) is most likely a product of mis-priming during amplification or sequencing, since bases 1–64 and 10–64 are 97% and 100% identical to the complement of a later segment of the sequence (bases 332–395), respectively. Removing this likely erroneous segment brings this sequence into agreement with our observations and those of Aoki et al. (2017), so it can be concluded that the 5′UTR for chicken PYY is approximately 38-39nt in length and the likely TSS is at genomic position chrUn_NT_464349v1:4497 (galGal5).

The structure of chicken PYY ligand differs from mammalian PYY, offering a unique opportunity to study the evolution of the ligand in tetrapods, and the effects of proteolytic cleavage on receptor specificity and action *in vivo*. Mammalian PYY_1-36_ undergoes cleavage to PYY_3-36_ by the action of dipeptidyl peptidase IV (DPP4). This reaction apparently confers the anorexigenic effect of the PYY ligand in rats by altering its receptor binding preference, whereas PYY_1-36_ has no effect on food intake in this species when DPP4 is inhibited (Unniappan et al., 2006). The primary structure of goldfish PYY is distinct from mammalian PYY, meaning it is insusceptible to DPP4 cleavage and PYY_3-36_ does not occur naturally in goldfish. Rat PYY_3-36_ has been shown to lack anorexigenic effects in goldfish, however goldfish PYY_1-36_ does attenuate food intake (Gonzalez and Unniappan, 2016), suggesting that the ligands and receptors have coevolved in each of these species, with anorectic signalling most likely by PYY_1-36_ via the Y1 receptor in goldfish and by PYY_3-36_ via the Y2 receptor in rats. Chicken PYY_1-37_ (as previously described) and quail PYY_1-37_ appear similar to goldfish PYY in their insusceptibility to DPP4 cleavage, suggesting that a role for DPP4 in posttranslational processing of mammalian PYY might have arisen relatively recently in the vertebrate evolutionary timeline, or has subsequently been lost in galliformes. The anorexigenic effects of chicken PYY_1-37_ and goldfish PYY_1-36_ remain dependent on origin at the peripheral side of the blood-brain barrier as the behavioural effect of PP-fold peptides within the brain is known to be functionally opposite (as classically attributed to NPY). Furthering previous work (Bromee et al., 2006, Gao et al., 2017, He et al., 2016, Holmberg et al., 2002, Lundell et al., 2002, Salaneck et al., 2000) to comprehensively define the roles and receptor preferences of avian PYY might offer insight into the evolution of PYY action in higher vertebrates more closely related to mammals.

The distribution of chicken PYY expression is different from mammals, with intestinal transcripts most abundant around the mid- to distal jejunum whereas peak mammalian intestinal PYY expression is thought to be in the distal ileum and large intestine (Miyachi et al., 1986, Pezeshki et al., 2012). Normalisation to reference genes suggested pancreatic expression is in fact higher than the intestinal peak in chickens and we surmise that the relatively high transcriptional activity of the pancreas (see Section 3.2) might have concealed the extent of PYY expression without reference gene normalisation. Incidentally, we also quantified PYY expression by qPCR across a tissue panel of a genetically distinct commercial brown layer line (Lohmann Classic) and found agreement between the distributional landscapes of PYY in broilers and layers, with the pancreas being the most prominent source of PYY mRNA (layer data not shown). The identification of the pancreas as the major site of PYY expression in chickens is contrary to the dogma that PYY is primarily of intestinal origin, but in keeping with recently-published chicken data (Gao et al., 2017). This is interesting because the first identified action of mammalian PYY was to regulate pancreatic function (Tatemoto, 1982), and since then PYY has been shown to mediate glucose homeostasis by paracrine activity in pancreatic islets (Boey et al., 2007, Ramracheya et al., 2016). In fact, specific targeting to the Y_2_ receptor – which seems to enhance the satiety effect of PYY in mammals – is dependent on processing of PYY_1-36_ to PYY_3-36_ by DPP4, but an orthologous cleavage is not known to exist for avian PYY_1-37_. These distinct capacities of the ligand raise further questions about the possible tissue of origin-dependence of PYY action *in vivo* and additional work is needed to investigate the precise effects of PYY on energy homeostasis in birds.

PYY and PPY expression levels were not dependent on the region of the pancreas sampled in young chicks (2wk), however both were significantly more highly expressed in the tail (splenic) than the head (duodenal) region of the pancreas in more mature birds (12wk), evidencing an ontogenic change in regional distribution of pancreatic PP-fold peptide expression in chickens. Ontogenic changes in expression levels of these peptides across many tissues have been identified in rats (El-Salhy et al., 1983, Sandström and El-Salhy, 2002), but the conservation and significance of these changes is poorly understood. Gradient distribution of pancreatic expression has also been noted in rats (Ekblad and Sundler, 2002) in which PPY and PYY were inversely correlated, in contrast to our findings in chickens where their gradients are directly correlated, at least at adolescence. Although we were unable to positively identify specific cell types expressing PP-fold peptide mRNA in chicken, we note that our results are in keeping with the observation of greatest islet abundance in the pancreas splenic half in mammals, and the association of PP-fold peptide synthesis with islets in vertebrates (Wang et al., 2013).

The dynamic regulation of pancreatic PYY (but not PPY) expression under short-term fasting and re-feeding suggests that pancreatic PYY may act as a short-term satiety signal in chickens, whereas PPY likely does not. Under long-term quantitative restriction of broiler-breeders with feeding once per day, the pattern of pancreatic PYY expression throughout the day was dependent on hunger/satiety level, as inferred by considering time until and since feeding, and whether the birds were active or sedentary for periods following feeding. To this end, birds fed a commercial restricted daily ration in the morning (Ram) experienced peak expression around seven hours after feeding which diminished within 16 h, whereas no acute increase in PYY expression was detected in birds fed the same ration in the afternoon (Rpm). This is possibly because afternoon-fed birds were in a state of low energy expenditure (sleep) soon after feeding and therefore have different immediate energy requirements. In humans, the duration of gastrointestinal hormonal response to feeding is attenuated during sleep (Soffer et al., 1997) and a similar attenuation could explain the apparent stability of PYY in the Rpm group of the long-term nutritional state experiment. The interaction of time of feeding and satiety hormone response is an interesting phenomenon which demands further study. The broiler breeder-type chickens used here are routinely food restricted in commercial production, leading to welfare concerns over hunger, and altering the time of feeding might offer a relatively easy management step to improve welfare. The comparative reduction in pancreatic PYY expression in food restricted birds was not attenuated by addition of dietary fibre (ispaghula husk) to the restricted ration, suggesting that pancreatic PYY expression is responsive to nutrient uptake, not merely physical gut fill. It is known that inclusion of small amounts of insoluble fibre aids digestibility in chickens (Hetland et al., 2003), but in this case birds fed relatively large amounts of ispaghula husk expressed pancreatic PYY at an almost-significantly lower level than restricted counterparts. This is possibly an artefact of the reduced speed with which birds consumed their ration or a negative effect of mucilaginous ispaghula fibre on digestibility.

The relative abundance and functional regulation of pancreatic PYY leads us to conclude that the pancreas is likely to be the major source of circulating PYY in chickens and that future studies of the roles of peripheral PYY and PPY in the energy balance of chickens should therefore focus on the pancreas. We have demonstrated that regional distribution of pancreatic PYY and PPY expression alters with age, and that pancreatic PYY expression is responsive to short-term nutritional state and is upregulated in satiety whereas PPY expression seems dependent on longer-term energy status. These proposed roles are contrary to the historical implication of PPY as simply a short-term satiety factor in chickens (Johnson and Hazelwood, 1982). It is noteworthy that the antibody used to quantify PPY was developed before the discovery of PYY (Tatemoto, 1982), meaning that possible cross-reactivity could not be assessed. It must however be conceded that the data herein represent only mRNA expression of chicken PYY and PPY, which might exhibit lagged response relative to expulsion of pancreatic peptide stocks or otherwise misrepresent the peptide output of expressing cells. The fluctuation of PPY in the AL group of the long-term nutritional state experiment (Section 2.2.3) implies that PPY expression is capable of significant short-term change, but this seems dependent on birds’ longer-term energy state. Additional work is therefore required to assess further the responsiveness of circulating chicken PYY and PPY peptides to feeding, and the effects of peripheral administration of exogenous PYY and PPY individually. Such work would be particularly valuable if it could complement characterisation of central PP-fold peptide activity. The unique attributes of avian PYY compared to mammalian PYY – namely exemption from DPP4 digestion and the chicken’s distinct pattern of dynamic expression – suggest that a comprehensive understanding of avian PP-fold peptides will contribute to the wider appreciation of the evolving roles of this family of molecules in higher vertebrate food intake and metabolism.
